# Moderation modelling of COVID-19 digital health literacy and sense of coherence across subjective social class and age among university students in Ghana

**DOI:** 10.1186/s40359-023-01334-9

**Published:** 2023-10-16

**Authors:** Isaac Amoako, Medina Srem-Sai, Frank Quansah, Stephen Anin, Edmond Kwesi Agormedah, John Elvis Hagan Jnr

**Affiliations:** 1 Department of Education, Atebubu College of Education, Bono East, Ghana; 2 Department of Interdisciplinary Studies, Akenten Appiah-Minka University of Skills Training and Entrepreneurial Development, P. O. Box 1277, Kumasi, Ghana; 3 Department of Health, Physical Education, Recreation and Sports, University of Education, P. O. Box 25, Winneba, Ghana; 4 Department of Educational Foundations, University of Education, P. O. Box 25, Winneba, Ghana; 5 Department of Industrial and Health Sciences, Faculty of Applied Sciences, Takoradi Technical University, P.O. Box 256, Takoradi, WS000 Ghana; 6 Department of Business & Social Sciences Education, University of Cape Coast, PMB, Cape Coast, Ghana; 7 Department of Health, Physical Education and Recreation, University of Cape Coast, PMB, Cape Coast, Ghana; 8 Neurocognition and Action-Biomechanics-Research Group, Faculty of Psychology and Sports Science, Bielefeld University, Postfach 10 01 31, 33501 Bielefeld, Germany

**Keywords:** Age, Computer literacy, Coronavirus, Coping, Sense of coherence, Subjective social class

## Abstract

**Background:**

The study assessed the moderation modelling of digital health literacy and sense of coherence across subjective social class and age among university students in Ghana during the COVID-19 pandemic.

**Methods:**

A total of 1160 students were conveniently sampled from two universities namely, the University of Education, Winneba and University of Cape Coast, using the descriptive cross-sectional survey design. Preliminary analysis was performed using descriptive statistics, whilst multivariate multiple regression and moderation analyses (Haye’s Model) were employed to analyze the main data.

**Results:**

The study revealed that COVID-19 digital health literacy is directly and positively associated with sense of coherence among university students. Further, higher subjective social class positively and strongly moderated the relationship between COVID-19 digital health literacy and sense of coherence among university students. Additionally, the relationship between COVID-19 digital health literacy and sense of coherence was indirectly prominent among relatively older university students than younger ones.

**Conclusions:**

The findings have implications for university management/authorities and public health agencies to organize effective orientation and self-management training programmes for university students.

## Introduction

The infamous COVID-19 pandemic triggered the continuous influx of a wide range of information on the internet and/or other social media platforms. The plethora of complex and sometimes, conflicting information on the viral disease generated uncertainty, panic, anxiety, and other mental health challenges, which led to poor quality of life and well-being [1–7]. Given this high influx of information, the concept of health literacy has become essential in the management of infectious diseases, including coronavirus and helps reduce its negative impact on individual and societal health [7]. Health literacy serves as catalyst in achieving universal health and wellbeing as captured by the Sustainable Development Goal 3.

Health literacy embodies one’s capacity to access, understand, appraise, and apply information to make informed decisions to promote and improve personal and community health through effective health decisions [8–11]. Existing empirical evidence suggests that individuals with low health literacy have low level of awareness and exhibit less protective behaviours, which increases their risk of COVID-19 infection [12–18]. An extra dimension of health literacy, the ability to access and process health information from electronic platforms, referred to as digital health literacy (DHL) [19], is identified as a significant buffer during the COVID-19 pandemic [20, 21], particularly due to the increased reliance on information from social media and other internet sources during the pandemic among young people [22]. Just like health literacy, DHL has also been negatively linked to mental health outcomes [23, 24]. Given the relevance of DHL amidst the pandemic, the concept of DHL was modified to COVID-19 DHL to refer to DHL during the COVID-19 pandemic.

Drawing from the perspective of Aaron Antonovsky’s salutogenic model, a person’s ability to comprehend and incorporate (comprehensibility), deal with (manageability) and make sense (meaningfulness) of an experience or disease because of information drawn from multiple sources, could also determine an individual’s likelihood to effectively cope with a health situation or disease [25–27]. The salutogenic model offers an interesting twist to the link between COVID-19 DHL and well-being; demonstrating that high level of DHL does not directly lead to better mental health outcomes, but boost individuals’ sense of coherence (SOC) – an ability to adapt when confronted with adversities or challenges [28]. The model further stresses that DHL serve as generalized resistance and specific resources to improve and promote an individual’s SOC for mitigating potential life adversities [26].

Except for the argument put forward by the salutogenic model concerning the relationship between DHL and SOC, very little is known about the DHL-SOC association. It is only recently that Leung and colleagues [20] confirmed the existence of a positive DHL-SOC relationship among older adults in China, Philippines and Singapore during the COVID-19 pandemic. However, what is known is the substantial negative connection between SOC and mental health problems in young populations [29–31]. A more intriguing discourse of the SOC-mental health link was demonstrated in a systematic review by da-Silva-Domingues et al. [29] which revealed that low level of SOC was associated with risky health-related behaviours such as poor oral health, illegal substance usage, inadequate rest pattern, poor eating habits, increased alcohol and tobacco use, increased time for playing computer games and reduced levels of physical activity. Having established that SOC predicts mental health outcomes, understanding how DHL predicts SOC is very critical in situations like COVID-19 pandemic where misinformation was found to be rampant, particularly, on social media [32]. This study has become necessary because especially when studies in Ghana have shown that youth with little COVID-19 DHL relied largely on non-reviewed media outlets [33]. Importantly, research into younger age groups is sparse, even though, these life phases are critical toward the development and shaping of SOC [34]. Hence, it is necessary to investigate the phenomenon within the African context, since the sub-region is noted to have the youngest population in the world [35]. Since young people make use of information, DHL and demographic characteristics may help inform relevant public health policies within the sub-region.

While DHL could be an essential resource for developing one’s SOC, the relationship could also be explained by one’s socio-demographic profile such as subjective social class (SCC) and age. Subjective Social Class (SSC), refers to a subjective measure of personal societal positioning or measure of one’s perceived social standing relative to a given social group, is predictive of different health and well-being outcomes [36, 37]. Based on Antonovsky’s salutogenic theory, an individual’s SSC (an indicator of socio-economic status) may determine his or her SOC (e.g., manageability - feeling self-assured that one possesses the required resources (e.g., financial) to meet life demands or stressors) [25, 27, 38]. Thus, the strength of an individual’s SOC somewhat depends on the availability of general resistance resources [27, 39], that is one’s SSC. For example, Kraus [40] indicated that having sufficient family income or financial resources may facilitate opportunities for activities that are perceived as healthy, meaningful, relevant, and enjoyable.

The fundamental cause theory also stipulates that one’s SSC also makes reference to the role of income in the development of DHL through diverse platforms, which in turn, positively predict SOC. Hence, social inequality such as income inequity may be related to the disparity in SOC. This assumption may not only be connected to individuals with low income but also because of the health benefits or satisfaction enjoyed by those with high income [41, 42]. Specifically, economic insecurity could cause distress and elicit poor coping strategies against challenging events like COVID-19 pandemic because of limited resources to manage daily needs [43]. Thus, together with DHL, financial stability could reinforce one’s SOC to promote optimal well-being during the pandemic period [20]. Consequently, SSC can help explain the relationship between DHL and SOC [44].

Previous studies have also documented that an individual’s age can predict the use of digital platforms (e.g., Facebook, YouTube, and Twitter) in search of health information during the COVID-19 pandemic period [24]. Some studies have proven that young adults below 30 years showed greater use of digital media during the COVID-19 pandemic than people who are 30 years and older [45, 46]. Using university students from Spain, Puerto Rico, and Ecuador, Rivadeneira et al. [24] established that DHL was directly and significantly associated with age. In a scoping review, Wang and Laun [47] showed that younger adults with a high socioeconomic status tend to have adequate/high DHL compared to older adults. Additionally, McGee et al. [48] found that older adults with strong manageability, a sub-dimension of SOC, had better mental health than their counterparts with poor manageability under high conditions of stress. Similarly, Coutinho and Heimer [49] found that adolescents with low SOC were more likely to exhibit anxiety and depressive symptoms whereas individuals with high SOC had higher odds of having a good quality of life.

Given the distinct distribution patterns of DHL and SOC across different social class and age brackets, these variables may play useful roles in the DHL-SOC relationship among university students. University students were used as a reference group in the current study because they represent a cohort with unique characteristics relative to the frequent usage of digital platforms or web-based technologies for diverse information and communication pertaining to studies and other needs [7, 18, 50, 51]. According to Zakar et al. [7], given the wide range of information that needs to be censored by university students, extracting useful information remains a huge challenge, especially in Ghana where availability, accessibility, and utilization of digital technologies in higher educational institutions are still constrained by various factors. These dynamics could impact students’ SOC and ultimately, their mental health. This study deepens the discussion on the determinants of SOC by focusing on the roles played by COVID-19 DHL, SSC and age. The findings of this study may be useful to tertiary education institutions in Ghana toward developing policies that enhance the DHL of students to help mitigate potentially adverse health outcomes. This study examined the moderating effect of SSC and age in the relationship between COVID-19 DHL and SOC of selected university students in Ghana. Specifically, the study sought to:

1. establish the link between COVID-19 DHL and SOC.
2. examine the relationship between COVID-19 DHL and SOC across SSC.
3. assess the relationship between COVID-19 DHL and SOC across ages.

**Fig. 1:**
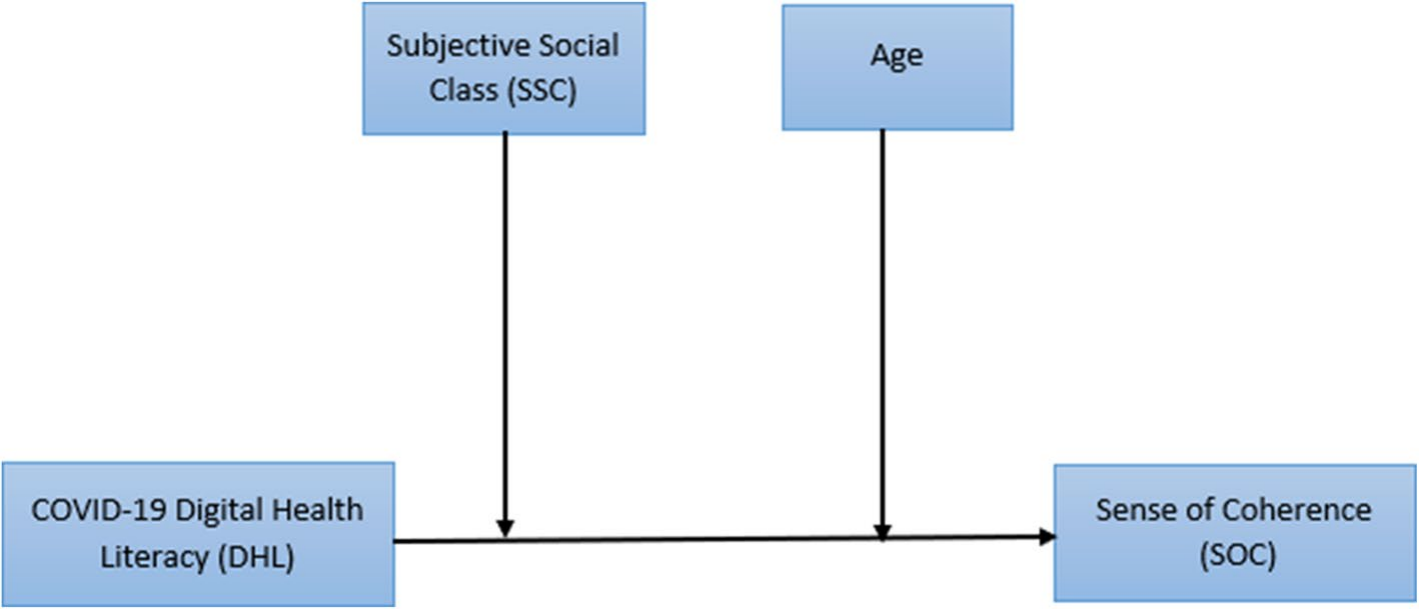
Conceptual framework showing the moderation effect of SSC and age in the relation between COVID-19 DHL and SOC

Based on the existing literature review, a conceptual model was developed to guide the study (see Fig. 1.)

## Methods and materials

### Study design and participants

The descriptive cross-sectional survey design was employed to conveniently sample 1160 university students from two public universities in Ghana. Only regular students (both postgraduate and undergraduate) were engaged for the study. All sandwich and distance students were excluded because they were not available at the time of conducting the study. Using the lecturers as the point of contact, the researchers visited the lecture halls of those instructors who had consented for their class to be used. In each class, students who indicated that they were available and willing to participate in the research were given the instrument to respond. The use of the convenience sampling strategy became necessary to use since the tensions around COVID-19 was still present and schools had just resumed after over 10 months of lockdown. The challenge was that several students were anxious and uncertain in terms participating in the study [5]. Hence, the researchers obtained 1,160 responses, although the target was to administer the questionnaire to 2,100 students which represents 5% of the population.

The majority (*n =* 965, 83.2%) of the students were undergraduate students, while 8% were postgraduate students (*n =* 195, 16.8%). Moreover, most of the participants were males 72% (*n* = 834) while 28% were females (*n* = 325). The ages of the participants ranged from 18 to 42 years. For this study, students of all bachelor’s or master’s degree majors were allowed to participate based on their availability. Because the study took place in a post-COVID-19 period with less restrictive protocols, participants were allowed to respond to survey questions through the face-to-face medium. However, relevant protocols such as social distancing and regular sanitizing were adhered to. There was no case of non-response.

### Variable measurements

In this study, COVID-19 DHL served as a predictor variable and SOC was used as a criterion variable. Both SSC and age were used as moderators. Also, sex of participants, the number of semesters spent on campus, and studying level of the respondents were used as control variables.

### Predictor variable

#### COVID-19 Digital Health literacy (DHL)

COVID-19 Digital health literacy (DHL), in this study, is conceptualised as the ability to seek, discover, understand, and critically scrutinise health information from electronic sources and apply the knowledge gained to solve a health problem during the COVID-19 pandemic [52]. With the original DHL scale developed by van Der Vaart and Drossaert [23], modified to the COVID-19 context by Dadaczynski et al. [53] and validated in Ghana [54], this study adopted the COVID-19 DHL scale for the measurement of COVID-19-related DHL of the university students. The latest version of the instrument has 12 items across four dimensions, namely, information searching, self-generated content, reliability, and determining relevance. The response format of the instrument was a 4-point Likert type, with the highest score of 4 representing the “very easy” response category and the lowest score of 1 for “very difficult" response option. This response format has been supported by the digital health literature [55, 56]. The following are samples of the items, that is, two items each on the four dimensions respectively: “*Make a choice from all the information you find*?“; “*Use the proper words or search query to find the information you are looking for*?“; “*Clearly formulate your question or health-related worry*?“; “*Express your opinion, thoughts, or feelings in writing*?“; “*Decide whether the information is reliable or not*?“; “*Check different websites to see whether they provide the same information*?“; “*Decide if the information you found is applicable to you*?“; and “*Apply the information you found in your daily life*?“ The obtained reliability estimate (i.e., using omega ω) was 0.76 for information searching, 0.79 for self-generated content, 0.78 for reliability and 0.72 for determining relevance. The Omega estimate is noted to be superior to the traditional Cronbach Alpha. Tau-variance calculations are involved in the former, whereas the latter’s estimation is based on factor loading [57].

### Criterion variable

#### Sense Of Coherence (SOC)

The SOC reflects the general orientation that people have the feeling of confidence to cope with challenges and stressful situations was the construct under consideration in this study, particularly during the COVID-19 era. This construct was measured using the SoC-9 scale by Schumacher et al. [58]. The SoC-9 scale is divided into three dimensions: meaningfulness (4 items), manageability (3 items), and comprehension (2 items). The response format of the scale is a 7-point Likert type ranging from 1 to 7. The total global score of the scale ranges from 9 to 63, and a higher score indicates a stronger SoC [58]. For the meaningfulness dimension, the following were some of the items: “*Caring about what goes on*” and “*View of life*.“ Some of the items on the manageability dimension also included “*Feeling of cooperation*” and “*Discontent of other people*,“ whereas “*Feeling of being understood*” and “*Feeling of knowing people*” were some of the items on the comprehensibility dimension. The original Cronbach Alpha reliability index reported ranged from .73 to .75. However, this study’s reliability calculated with Omega-ω produced .73 to .81.

### Moderator variables

#### Subjective Social Class (SSC)

Subjective social class (SSC) reflects the relative perception that individuals have of their place in the social hierarchy [59]. The variable was measured using the MacArthur SSC scale based on a relative social comparison process. There are two versions of this scale, the society ladder, which reflects a global measure of subjective social status and the community ladder which talks about how individuals see themselves considering the community in which they live [60]. This study made use of the society ladder option to get a picture of how the students perceived their economic and educational status within society. The indicators used in this scale reflect income, education, and occupation. The preamble to the question is “*consider that the ladder that I am showing you represents the place that people occupy in society. At the top of this ladder are the people who have more money, more education, and better jobs (jobs with more recognition). At the bottom of the ladder are people who have less money, education, and worse jobs. Considering the living standards of the people in the neighbourhood. The higher you consider yourself on the ladder, the closer you will be to the people who are at the top of the ladder, and the lower, closer you will be to people who find themselves at the bottom. Where would you place yourself on this ladder*?” The scale is presented in a ladder format with 1 to 10 hierarchical steps. While the lower point is at 1, point 10 reflects the higher status. Scores ranging from 1 to 4 represent low SSC, 5 to 7 signify average SSC whereas 8 to 10 demonstrates high SSC. The Kappa statistics that reflected the reliability of the items was 0.81 which was deemed as good [61].

### Age

In the context of this research, age was measured from the chronological age perspective, which is characterized by the number of years from birth to a given date that the person is living [62]. Although the chronological age spans from the second, days, weeks, and months that a person has lived, we focused on the approximate years of the students at the time of data collection. Thus, the participants were required to provide their ages in approximated years by writing. For the purposes of the moderation analysis, participants who were between the ages of 18 and 25 years were considered young adults and those who were between 26 and 45 were classified as middle adults [63].

### Control variables

Three demographic variables were used as covariates in the analysis with the understanding they might have influence on any of the major variables. Hence, we controlled for the sex of participants, number of semesters spent on campus, and studying level of the respondents (i.e., undergraduate, and postgraduate). Since sex and studying level were categorical variables, dummies were created and used for the regression-based analyses performed. For sex, males were compared to females (reference group) whereas undergraduate students were also compared with postgraduate students (reference group). The number of semesters spent on campus variable was treated as continuous.

### Data collection procedures

The study received ethical clearance from the Ethical Review Board (IRB) of the University of Education Winneba, Ghana, with reference number DAA/ P.1/Vol.1/39. Further, approval was sought from the Heads of Departments from which students were selected. The data were gathered from the lecture halls of the universities after the necessary arrangements had been made with the lecturers whose lecture time would be used. To ensure that the students were not vulnerable in terms of being indirectly coerced, the lecturers were made to excuse the class for the data collection exercise. The questionnaires were distributed to the participants by the study’s researchers. Prior to the administration, the researchers established rapport with the participants. As part of the rapport creation, participants were briefed about the details of the investigation, particularly on the purpose of the work and the need to participate in the investigation. Participants were assured that their responses would be kept confidential and that to ensure anonymity, anything that identifies them, such as names, index numbers, etc., should not be provided on the questionnaire. Voluntary participation awareness was also extended to the participants, such that, whenever they so desired, they could opt out of the study without any consequences. Moreover, informed consent forms were made available to each participants to sign to indicate their willingness to participate in the study. Responding to the survey instrument took participants about 30 min. The whole data collection exercise lasted for eight weeks. Even though the study took place in the post-COVID-19 era, some important protocols such as hand washing, social distancing, and sanitising regularly were adhered to strictly in order to protect participants.

### Data analysis plan

Analyzing the data collected for this study was done using varied statistical approaches. The analysis was conducted using SPSS (version 25) computer programming statistical software. First, the data were screened and cleaned for data entry errors and outliers of the variables. There were no errors during the data coding process. Data analysis for the study was structured in different phases based on the objectives of the study. Prior to the major analysis, Harman’s single factor test was performed via exploratory factor analysis in order to understand the common method bias associated with this research. The analysis showed that the total variance extracted by a single factor was 14.63% which is less than 50%. The preliminary analysis examined the mean, standard deviation, skewness, and kurtosis for both continuous data. The first objective which sought to investigate the relationship between COVID-19 DHL and SoC was addressed by performing multivariate multiple regression analysis. Objective two, which aimed to examine the relationship between COVID-19 DHL and SOC across SSC was analysed using moderation analysis (Haye’s model 1) using PROCESS MACRO software by Andrews Hayes [64]. Similarly, moderation analysis (Hayes model 1) was also performed to investigate the relationship between COVID-19 DHL and SOC across ages. In all the inferential analyses, the bootstrapping procedure with 5000 bootstrap samples corrected confidence intervals were used for the parameter estimation [65].

## Results

### Preliminary analyses

Table 1 provides the mean, standard deviation, skewness, and kurtosis of the variables of the study.

**Table 1:** Mean, SD, skewness, and kurtosis of the variables

Main Construct	Variables	Mean	SD	Skewness	Kurtosis
COVID-19 DHL Dimensions	Information searching	2.09	.819	.410	−.725
	Self-generated content	2.14	.787	.535	−.380
	Evaluating reliability	2.26	.753	.346	−.642
	Determining relevance	2.14	.822	.308	−.827
SoC Dimensions	Meaningfulness	3.07	.910	.891	.702
	Manageability	4.67	1.229	−.507	.838
	Comprehensibility	2.85	1.554	.022	-1.004
Subjective Social Status	Subjective social class	4.20	1.252	1.072	2.284
Age	Age	26.88	5.527	.513	−.468

The descriptive statistics of the data showed that the COVID-19 DHL dimensions had values ranging from 2.09 to 2.26, with the students exhibiting higher skills in evaluating the reliability of information searched and lower skills in information searching. The SOC dimensions had scores ranging from 2.85 (for comprehensibility) to 4.67 (for manageability). The mean SSC ranking of the participants was 4.20 with a standard deviation of 1.252. The mean age was 26.88 with a standard deviation of 5.527. All skewness and kurtosis values for the values were acceptable and within the established ranges [66, 67].

#### Relationship between COVID-19 DHL and SOC

The study examined the association between COVID-19 DHL and SOC using multivariate multiple regression analysis. Due to the multivariate nature, a stringent alpha was set from .05 to .017 based on the number of criterion variables. The results are presented in Table 2.

**Table 2:** Parameter estimate of the relationship between COVID-19 DHL and SOC

Dependent Variable	Parameter	B	Std. Error	t	Sig.	95% CI	
						LLCI	ULCI
Meaningfulness	Intercept	2.552	.274	9.306	.000	2.014	3.090
	Information searching	.169	.062	2.719	.007	.291	.047
	Self-generated content	.240	.063	3.775	.000	.115	.364
	Evaluating reliability	.095	.057	1.663	.097	− .017	.207
	Determining relevance	.107	.041	2.602	.009	.026	.188
	Sex [Male]	− .026	.059	− .447	.655	− .143	.090
	Study Level [Undergraduate]	− .113	.098	-1.156	.248	− .306	.079
	Number of semesters	.056	.020	2.764	.006	.016	.096
Manageability	Intercept	4.643	.376	12.362	.000	3.906	5.380
	Information searching	− .058	.085	− .683	.495	− .225	.109
	Self-generated content	.109	.087	1.252	.211	− .062	.280
	Evaluating reliability	− .029	.078	− .370	.712	− .183	.125
	Determining relevance	.202	.056	3.587	.000	.092	.313
	Sex [Male]	− .269	.081	-3.306	.001	− .428	− .109
	Study Level[Undergraduate]	− .017	.134	− .127	.899	− .280	.246
	Number of semesters	.014	.028	.511	.610	− .041	.069
Comprehensibility	Intercept	3.232	.448	7.223	.000	2.354	4.111
	Information searching	− .161	.101	-1.588	.112	− .360	.038
	Self-generated content	− .208	.104	-2.003	.045	− .411	− .004
	Evaluating reliability	.627	.093	6.719	.000	.444	.811
	Determining relevance	.427	.067	6.360	.000	.295	.559
	Sex [Male]	− .190	.097	-1.963	.050	− .380	-4.763
	Study Level [Undergraduate]	− .487	.160	-3.047	.002	− .801	− .174
	Number of semesters	− .175	.033	-5.247	.000	− .240	− .109

The outcome of the analysis showed that information searching [*B =* .169, *t =* 2.719, *CI* (.291, .047)], self-generated content [*B =* .240, *t =* 3.775, *CI*(.115, .364)], and determining information relevance [*B =* .107, *t =* 2.602, *CI* (.026, .188)] competencies of COVID-19 DHL significantly predicted meaningfulness, suggesting that an individual student’s ability to search for information, generate the content desired with appropriate keywords and determine the relevance of COVID-19 information helps him/her to unearth meaning in life during COVID-19 related challenges. Similarly, determining information relevance competencies, *B =* .202, *t =* 3.587, *CI*(.092, .313) also significantly predicted manageability. The result further suggested that students’ ability to evaluate the reliability of the COVID-19 information [*B =* .627, *t =* 6.719, *CI* (.444, .811)]; and determine information relevance of the COVID-19 related information taken [*B =* .427, *t =* 6.360, *CI* (.295, .559)] define their perception of their environment as structured and predictable. In effect, COVID-19 DHL played a significant role in students’ general view of coping with the challenges and stress posed by the COVID-19 pandemic.

#### Relationship between COVID-19 DHL and SOC across SSC

The relationship between COVID-19 DHL and SOC across SSC of the students was explored. The details are shown in Table 3.

**Table 3:** Moderation effect of SSC in the relationship between DHL and SOC

Predictor		B	SE	*T*	p	BootLLCI	BootULCI
Information search (ISH)	Constant	3.109	.221	14.079	.000	2.675	3.543
	ISH	− .179	.087	-2.071	.039	− .349	− .009
	SSC	.099	.057	1.730	.084	− .013	.212
	ISH*SSC	.039	.019	2.139	.033	.003	.076
	Sex [Male]	− .190	.048	-3.938	.000	− .285	− .095
	Study Level [Undergraduate]	− .186	.078	-2.367	.018	− .340	− .032
	Number of semesters	− .034	.016	-2.059	.040	− .066	− .002
	SGC	.005	.052	.102	.919	− .096	.107
	DRL	.188	.047	4.020	.000	.096	.280
	DRV	.180	.036	5.032	.000	.110	.251
Self-generated content (SGC)	Constant	3.303	.228	14.460	.000	2.855	3.751
	SGC	− .2032	.0892	-2.278	.023	− .378	− .028
	SSC	.0243	.0591	.411	.681	− .092	.140
	SGC*SSC	.0577	.0191	3.026	.003	.020	.095
	Sex [Male]	− .185	.048	-3.829	.000	− .280	− .090
	Study Level [Undergraduate]	− .183	.078	-2.336	.020	− .337	− .029
	Number of semesters	− .035	.016	-2.131	.033	− .067	− .003
	ISH	− .167	.050	-3.316	.001	− .266	− .068
	DRL	.182	.047	3.884	.000	.090	.274
	DRV	.176	.036	4.895	.000	.106	.247
Determining reliability (DRL)	Constant	2.968	.2376	12.494	.000	2.503	3.435
	DRL	.002	.090	.017	.987	− .175	.178
	SSC	.053	.061	.862	.389	− .068	.174
	DRL*SSC	.033	.019	1.693	.091	− .005	.071
	Sex [Male]	.190	.048	3.950	.000	.285	.096
	Study Level [Undergraduate]	− .188	.078	-2.402	.016	− .342	− .035
	Number of semesters	− .034	.016	-2.061	.039	− .066	− .002
	ISH	− .164	.050	-3.260	.001	− .263	− .065
	SGC	.000	.052	.001	.999	− .102	.102
	DRV	.181	.036	5.065	.000	.111	.251
Determine relevance (DRV)	Constant	2.867	.205	13.962	.000	2.464	3.270
	DRL	.103	.080	1.287	.198	− .054	.261
	SSC	.050	.054	.924	.356	− .056	.156
	DRL*SSC	.024	.017	1.374	.170	− .010	.058
	Sex [Male]	− .191	.048	-3.966	.000	− .285	− .096
	Study Level [Undergraduate]	− .188	.078	-2.398	.017	− .342	− .034
	Number of semesters	− .034	.016	-2.095	.036	− .067	− .002
	ISH	− .167	.050	-3.320	.001	− .266	− .068
	SGC	− .001	.052	− .026	.980	− .104	.101
	DRL	.188	.047	4.019	.000	.096	.279

Model Summary: *R*^2^ = .1525; F(3, 1140) = 68.3542, *p* = .001. Criterion is Sense of Coherence (SOC), *SSC* Subjective social class, *B* unstandardized coefficient, *LLCI * Lower Limit Confidence Interval, *ULCI*  Upper Limit Confidence Interval, *SE* Standard Error; Asterisk (*) represent interaction sign

This objective examined the moderating effect of SSC on the relationship between COVID-19 DHL and SOC among university students. Results shown in Table 3 showed that SSC, *B* = 0.397, *SE* = .019, *BootCI* (.003, .076), had a significant positive moderating effect on the relationship between “information searching” and SOC. Likewise, SSC, *B* = .0577, *SE* = .0191, *Boot CI* (.020, .095), positively moderated the relationship between self-generated content on COVID-19 information and SOC. However, SSC did not significantly moderate the connection between SOC and their ability to evaluate the reliability of COVID-19-related information and/or determine information relevance of COVID-19-related information.

**Fig. 2:**
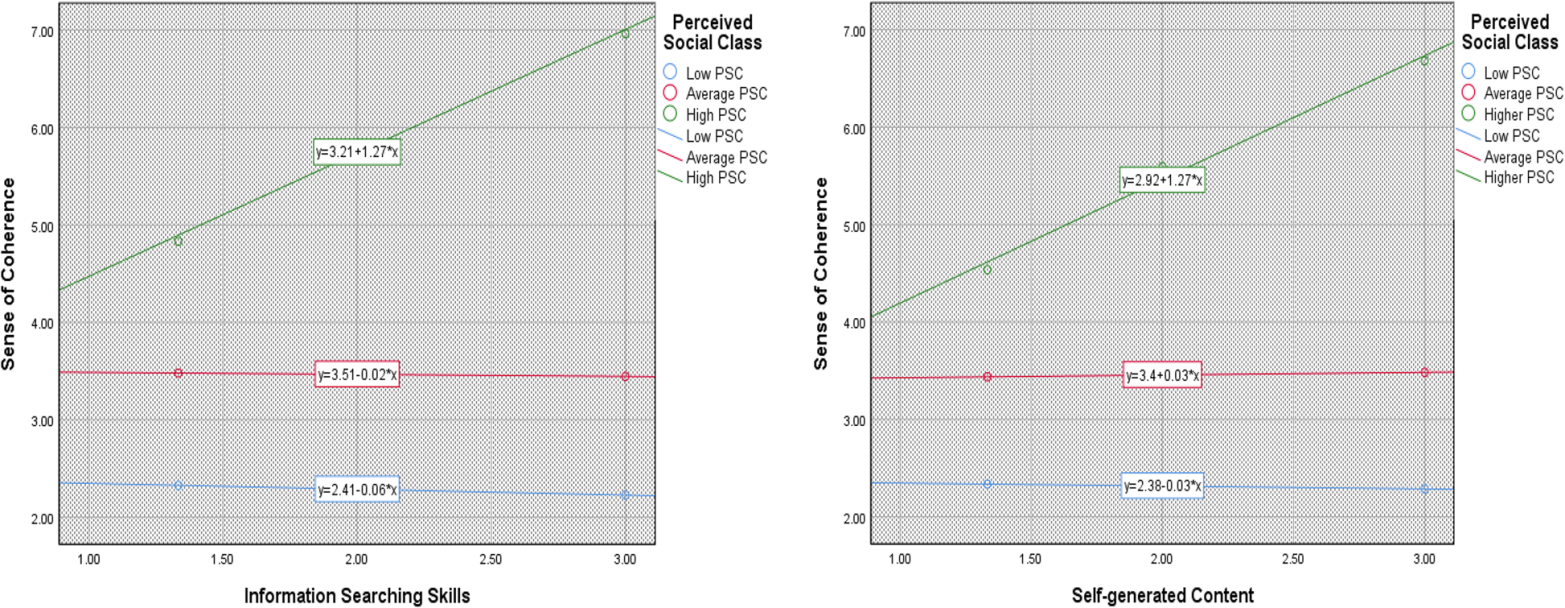
Probing interaction effect of SSC in the relationship between (**a**) information searching and SOC, and (**b**) self-generated content and SOC

From Fig. 2, the significant relationship between information searching abilities and SOC was positive and strong for those with higher SSC; hence, the steeper the slope for the line representing that group. Conversely, the association between the information-searching skill of COVID-19 DHL and SOC appeared slightly negative. Further, the link between self-generated content abilities and SOC was positive and strong for those with higher SSC compared to those with moderate/low SSC.

#### Relationship between DHL and SOC across age groups

The relationship between COVID-19 DHL and SOC across age groups of students was examined. A summary of the analysis is shown in Table 4.

**Table 4:** Moderation effect of age group in the relationship between COVID-19 DHL and SOC

Predictor		B	SE	T	p	BootLLCI	BootULCI
Information search (ISH)	Constant	3.879	.1909	20.3162	.0000	3.5044	4.2537
	ISH	.017	.0878	.1912	.8484	− .1554	.1890
	Age	− .546	.1207	-4.5198	.0000	− .7822	− .3087
	ISH*Age	.139	.054	2.561	.010	.032	.245
	Sex [Male]	− .112	.048	-2.322	.020	− .206	− .017
	Study Level [Undergraduate]	− .228	.078	-2.928	.003	− .381	− .075
	Number of semesters	− .022	.016	-1.356	.175	− .054	.010
	SGC	.034	.050	.667	.505	− .065	.133
	DRL	.240	.045	5.292	.000	.151	.329
	DRV	.242	.033	7.424	.000	.178	.306
Self-generated content (SGC)	Constant	3.765	.200	18.8650	.0000	3.3731	4.1562
	SGC	.066	.089	.7391	.4600	− .1094	.2416
	Age	− .525	.126	-4.1588	.0000	− .7728	− .2773
	SGC*Age	.129	.056	2.321	.020	.020	.238
	Sex [Male]	− .106	.048	-2.201	.028	− .201	− .012
	Study Level [Undergraduate]	− .220	.078	-2.819	.005	− .373	− .067
	Number of semesters	− .022	.017	-1.325	.185	− .054	.011
	ISH	.119	.050	2.380	.017	.216	.021
	DRL	.241	.045	5.310	.000	.152	.331
	DRV	.239	.033	7.323	.000	.175	.303
Determining reliability (DRL)	Constant	3.443	.216	15.916	.000	3.019	3.868
	Reliability	.2170	.0930	2.3340	.020	.035	.400
	Age	− .429	.137	-3.140	.002	− .696	− .161
	DRL*Age	.072	.058	1.247	.213	− .041	.185
	Sex [Male]	− .106	.048	-2.209	.027	− .201	− .012
	Study Level [Undergraduate]	− .223	.078	-2.857	.004	− .377	− .070
	Number of semesters	− .026	.016	-1.572	.116	− .058	.006
	ISH	− .110	.050	-2.213	.027	− .207	− .012
	SGC	.042	.051	.832	.405	− .057	.141
	DRV	.247	.033	7.515	.000	.182	.311
Determine relevance (DRV)	Constant	3.174	.192	16.509	.000	2.797	3.551
	DRV	.338	.085	3.964	.000	.171	.506
	Age	− .228	.119	-1.914	.056	− .460	.006
	DRV*Age	− .006	.052	− .109	.913	− .108	.097
	Sex [Male]	− .105	.048	-2.181	.029	− .200	− .011
	Study Level [Undergraduate]	− .213	.078	-2.727	.006	− .367	− .060
	Number of semesters	− .029	.016	-1.783	.075	− .061	.003
	ISH	− .106	.050	-2.142	.032	− .204	− .009
	SGC	.037	.051	.732	.464	− .062	.136
	DRL	.241	.046	5.243	.000	.151	.331

Model Summary: *R*^2^ = .0451; F(3, 1140) = 17.9427, *p* = .001. Criterion: Sense of Coherence (SOC), *B* unstandardized coefficient, *LLCI*  Lower Limit Confidence Interval, *ULCI*  Upper Limit Confidence Interval, *SE* Standard Error; Asterisk (*) represent interaction sign

In the first model, age was found as a significant moderator in the relationship between the information searching dimension of COVID-19 DHL and SOC, *B* = .139, *SE* = .054, *BootCI* (.032, .245) (see Table 4). Further, the age category was noted to significantly moderate the correlation between self-generated content of COVID-19 DHL and SOC, *B* = .129, *SE* = .056, *BootCI* (.020, .238). The significant moderation effect of age in the relationship of the models necessitated a post hoc analysis. The results of the post hoc analysis are shown in Table 5. Age failed to moderate the relationship between students’ ability to evaluate the reliability of COVID-19-related information and SOC, *B* = 072, *SE* = .058, *BootCI* (-.041, .185); as well as the relationship between determining information relevance of COVID-19-related information and SOC, *B* = − .006, *SE* = .052, *BootCI* (-.108, .097).

**Table 5:** Probing the moderating effect of age for information searching and self-generated content dimensions

	Age category	Effect	SE	t	P	LLCI	ULCI
ISH	Young adulthood	.155	.040	3.895	.000	.077	.234
	Middle adulthood	.294	.037	8.036	.000	.222	.365
SGC	Young adulthood	.195	.040	4.829	.000	.116	.275
	Middle adulthood	.324	.038	8.490	.000	.249	.399

The probing results, as indicated in Table 5 showed that the relationship between the “information searching” dimension of COVID-19 DHL and SOC, is significantly higher for middle-aged adults, *B =* .294, *SE =* .037, *BootCI* (.222, .365) than young adults, *B =* .155, *SE =* .040, *BootCI* (.077, .234). Regarding the self-generated content of COVID-19 DHL and SoC, the effect was greater for middle-aged adults, *B =* .324, *SE =* .038, *BootCI* (.249, .399) than young adults, *B =* .195, *SE =* .040, *BootCI* (.116, .275).

## Discussion

This study examined the moderating roles of age and SSC on the presumed relationship between four dimensions of COVID-19 DHL and three domains of SOC among university students. The association between COVID-19 DHL and SOC was premised and hypothesized in this study from Antonovsky’s salutogenic model that a person’s ability to comprehend, manage, and make sense of any disease or adverse health condition could determine his/her capacity to effectively adapt and cope with the situation (SOC). This presumed direct relationship was first examined from a multivariate multiple regression analysis of the various dimensions of DHL and SOC. It was inferred that the comprehension dimension of SOC was consistently predicted by evaluating reliability and determining relevance skills of COVID-19 DHL. Whereas the determining relevance dimension was positively predicted by the manageability (ability to deal with the COVID-19 pandemic) component of SOC, both evaluating reliability and determining relevance dimensions predicted meaningfulness (ability to make sense of COVID-19 information) domains of SOC. The varying strengths and directions in the relationships between the various domains of COVID-19 DHL and SOC point to the multidimensional complexity of these two constructs, and the possible influences of contextual moderator variables. Notwithstanding, the findings affirm generally that there is a direct relationship between COVID-19 DHL and SOC. The significantly strong and positive moderating role of higher SSC found in the relationship between the information searching and self-generated content dimensions of COVID-19 DHL and SOC suggests that one’s perceived social status (personal societal positioning or social standing relative to a given social group) was an important health-promoting and/or protective resource to ensure one’s health and SOC. The positive moderating role of higher age as a determinant of the positive relationship between DHL and SOC also contributes to unravelling a deeper understanding of why older students may fare better than younger students when faced with adverse health or medical conditions.

Studies conducted by Rivadeneira et al. [24] among Spanish-speaking university students, by Zakar et al. [7] among university students in Pakistan, and older adults (60 years or more) in three Asian countries [20] detected similar observations in the strong, positive, and direct associations between COVID-19 DHL and SOC. This consistency in the strength and direction of the correlation between COVID-19 DHL and SOC is supportive of the myriads of links inferred between DHL in general and the well-being of individuals when exposed to various health stressors [8, 19, 21, 23]. COVID-19 DHL as a potential preventive and/or protective factor against various forms of adverse psychosocial health outcomes such as depression, anxiety, and poor quality of life among others [6, 12] is likely to enhance one’s ability to search, understand, evaluate, and apply relevant health information [10], with its attendant benefits. The comprehensibility dimension of SOC (feeling of knowing people and being understood) was most consistently predicted by COVID-19 DHL suggesting that it is possibly more essential for university students to make sense of COVID-19, rather than view the health menace as something beyond one’s capacity to deal with [68].

No study so far has reported a significantly strong and positive moderating role of higher SSC as observed in the relationship between COVID-19 DHL and SOC in this study among university students. This is a novel finding. The lack of research on the moderating role of SSC on COVID-19 DHL and SOC among university students and other similar study settings, therefore, warrants more attention [20]. This enhancing indirect effect of higher SSC could be explained from the viewpoints of the fundamental cause theory, which supports the hypothesis of this study on the moderating role of SSC between COVID-19 DHL and SOC. The fundamental cause theory posits that health inequalities are strongly associated with the disparities in the socioeconomic status of individuals and populations in that the lack of, or inadequate financial resources could stifle the attainment of optimal health and wellbeing [69]. Accordingly, students with high socio-economic status can avoid poor health choices/conditions using the money, knowledge, and social networks at their disposal unlike individuals with low SES. Conversely, under-resourced students are more likely to face constraints in achieving high COVID-19 DHL and consequently increasing the propensity for untoward health outcomes and/or lower SOC during the COVID-19 pandemic. Given that high COVID-19 DHL is considered a general resistance resource in line with the salutogenic model [26, 27] and has been reported to be significantly associated with higher socioeconomic status (SES) and/or social class [37, 70, 71], the capacity of university students to experience higher SOC cannot be decoupled from their social class or SES as observed in a study among people living with HIV AIDS in Japan [72].

The statistically significant and positive moderating effect of age on the relationship between COVID-19 DHL and SOC observed in this study has also not been explored among university students or any other similar relatable context. With the knowledge that the African continent harbours a youthful population, this finding is particularly important for the African sub-region because knowledge of how young people could explore emerging technologies will be shared. It is public knowledge that young people, such as university students, are easily regarded as being proficient with digital technology and are called “digital natives”. However, some young university students’ may experience difficulties in finding, understanding, and utilising information [18]. The observed positive moderating role of higher SSC between COVID-19 DHL and SOC in this study among university students’ reiterates that the digital divide [73, 74] syndrome still persist within the African continent. The novel finding raises further awareness and empirical conversations around bridging the digital gap, sustainable wellbeing, and public health advocacy. Age as a non-modifiable risk factor has been explored in the promotion of health and wellbeing [75].

### Strengths and limitations

To the best of our knowledge, this is the first study to explore the moderating roles of age and SSC in the relationship between COVID-19 DHL and SOC for any population so far. This study, therefore, provides useful information for further research. The nonparametric resampling method (bootstrapping procedure) used in this study helped to overcome the limitations (bias-corrected parameter estimates) of the relatively small sample size of this study [65, 76].

The cross-sectional study data was collected using a non-probability sampling method which limits the representativeness of the university students recruited for the study population of interest. The research design used also precludes inference of causality between COVID-19 DHL and SOC. The variables studied were measured through self-reported instruments, which could have introduced some possible social desirability biases among the students’ responses, even though the COVID-19 DHL instrument has been validated in a similar setting among university students. Future researchers are encouraged to investigate DHL among other populations in line with sociocultural variables using combined methodologies (e.g., exploratory mixed-method approach).

### Practical implications

This study provides preliminary insights into the potential roles of age and SSC in the relationship between COVID-19 DHL and SOC as general resistance resources for developing resilience in times of adversity or disease experience. Educational authorities are encouraged to pay particular attention to younger students, especially freshmen and women compared to continuing students as they transition and integrate into their new learning environments. Resources needed to enhance the DHL of students ought to be provided to help mitigate potentially adverse health outcomes and/or sub-optimal SOC. Public health interventions such as self-management training programmes could serve as tools for health promotion for health-enhancing behaviours. This orientation could be organized for university students, especially freshmen to enhance their subjective perceptions of their social class [68].

## Conclusions

COVID-19 DHL is directly and positively associated with SOC among university students. Higher SSC positively and strongly moderates the relationship between COVID-19 DHL and SOC. The relationship between COVID-19 DHL and SOC is indirectly accentuated among relatively older university students than younger ones.

## Data Availability

Anonymized data is available upon reasonable request through the corresponding author.

## Glossary

COVID-19: Coronavirus
DHL: Digital health literacy
COVID-19 DHL: Coronavirus digital health literacy
SOC: Sense of coherence
SSC: Subjective social class
